# Systematic Analysis of the DNA Damage Response Network in Telomere Defective Budding Yeast

**DOI:** 10.1534/g3.117.042283

**Published:** 2017-05-25

**Authors:** Eva-Maria Holstein, Greg Ngo, Conor Lawless, Peter Banks, Matthew Greetham, Darren Wilkinson, David Lydall

**Affiliations:** * Institute for Cell and Molecular Biosciences, Newcastle University, Newcastle upon Tyne, NE2 4HH, United Kingdom; † Institute of Cancer and Genetics, School of Medicine, Cardiff University, CF14 4XN, United Kingdom; ‡ The Gurdon Institute and Department of Zoology, University of Cambridge, CB2 1QN, United Kingdom; § School of Mathematics and Statistics, Newcastle University, Newcastle upon Tyne, NE1 7RU, United Kingdom

**Keywords:** DNA damage, telomere, yeast

## Abstract

Functional telomeres are critically important to eukaryotic genetic stability. Scores of proteins and pathways are known to affect telomere function. Here, we report a series of related genome-wide genetic interaction screens performed on budding yeast cells with acute or chronic telomere defects. Genetic interactions were examined in cells defective in Cdc13 and Stn1, affecting two components of CST, a single stranded DNA (ssDNA) binding complex that binds telomeric DNA. For comparison, genetic interactions were also examined in cells with defects in Rfa3, affecting the major ssDNA binding protein, RPA, which has overlapping functions with CST at telomeres. In more complex experiments, genetic interactions were measured in cells lacking *EXO1* or *RAD9*, affecting different aspects of the DNA damage response, and containing a *cdc13-1* induced telomere defect. Comparing fitness profiles across these data sets helps build a picture of the specific responses to different types of dysfunctional telomeres. The experiments show that each context reveals different genetic interactions, consistent with the idea that each genetic defect causes distinct molecular defects. To help others engage with the large volumes of data, the data are made available via two interactive web-based tools: Profilyzer and DIXY. One particularly striking genetic interaction observed was that the *chk1∆* mutation improved fitness of *cdc13-1 exo1∆* cells more than other checkpoint mutations (*ddc1∆*, *rad9∆*, *rad17∆*, and *rad24∆*), whereas, in *cdc13-1* cells, the effects of all checkpoint mutations were similar. We show that this can be explained by Chk1 stimulating resection—a new function for Chk1 in the eukaryotic DNA damage response network.

The most important function of telomeres is to shield chromosome ends from being recognized as DNA double-strand breaks (DSBs). The DNA damage response (DDR) to dysfunctional telomeres strongly affects genome stability, ageing, and cancer (Gunes and Rudolph 2013; Artandi and DePinho 2010; Aubert and Lansdorp 2008; Blackburn *et al.* 2015). In budding yeast, the fitness of cells with defective telomeres can be increased, or decreased, by mutations affecting scores of different processes (Addinall *et al.* 2011). Analogous genetic interactions in human cells presumably affect ageing and cancer.

Two broadly different classes of protein bind telomeres (Figure 1A). Proteins that directly bind telomeric DNA of normal cells are generally important for physiological telomere function. A different set of proteins, principally components of the DDR network, binds defective telomeres, exemplified by those interacting at telomeres of *cdc13-1* mutants (Figure 1A). However, these two classes of protein are not distinct. For example, in most contexts, Ku, MRX, and Tel1 are considered DDR proteins, but, at telomeres, they protect from the DDR, and are important for maintaining normal telomere length (Wellinger and Zakian 2012; Lydall 2009).

**Figure 1:**
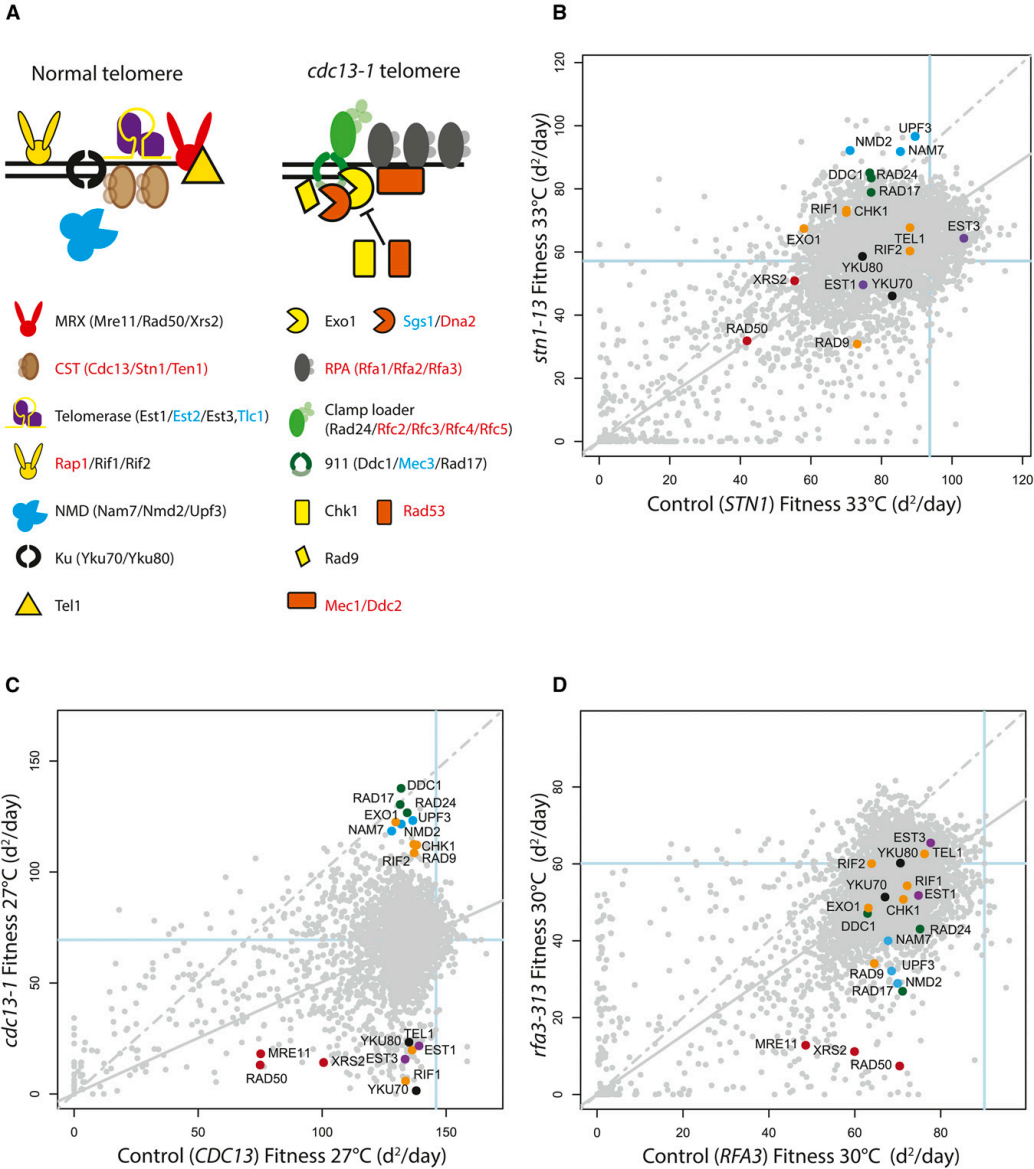
Genome-wide analysis of genetic interactions affecting the function of proteins that bind to ssDNA. (A) Molecules that interact at normal telomeres (left) or *cdc13-1* telomeres (right). Multi-component complexes are highlighted. Most proteins illustrated directly interact with telomeric DNA, and/or at DNA damage. The NMD complex affects telomere length, indirectly, at least in part by affecting the levels of Stn1 and Ten1. Black text refers to proteins removed by the 19 gene deletions highlighted in (B–D). Blue text labels gene products not shown in B–D. These are Mec3 and Tlc1, which were missing from the genome-wide knock out collection used, Est2 which behaved differently from its partners Est1 and Est3 in most screens, and Sgs1 which was comparatively unfit in most screens. Red text labels essential genes, which cannot be deleted. (B) Fitness plot showing genetic interactions between members of the yeast knockout collection and *stn1-13*. Each point summarizes the effect of *yfg∆* mutations on *STN1 lyp1∆* or *stn1-13* strain fitness at 33°. The colored points label gene deletions affecting proteins highlighted in (A). Fitness is measured as Maximum Doubling Rate × Maximum Doubling Potential (MDR × MDP, units are doublings squared per day, d^2^/day), as previously described (Addinall *et al.* 2011). The dashed gray line represents the line of equal fitness in both strain backgrounds, and solid gray is the predicted fitness assuming genetic independence. (C) Same as in (B) but in *CDC13 ura3∆* or *cdc13-1* backgrounds, and at 27°. (D) Same as in (B) but in *RFA3 lyp1∆* and *rfa3-313* contexts, and at 30°.

Dividing cells need to overcome the end replication problem to maintain telomere length and function. Defects in telomerase, MRX, NMD, Ku, or Tel1 cause short telomeres, while defects in Rif1 or Rif2 cause long telomeres (Lydall 2009; Wellinger and Zakian 2012). Many proteins that affect telomere length, are not critical to yeast telomere function, since they can be deleted and cells remain viable. In contrast, the CST complex, consisting of Cdc13, Stn1, and Ten1, is essential. CST binds telomeric single stranded DNA (ssDNA), and plays a critical role in telomere protection and telomerase recruitment (Wellinger and Zakian 2012). The analogous complex in plant and animal cells is encoded by *CTC1*, *STN1*, and *TEN1* (Bertuch and Lundblad 2006; Chen *et al.* 2012; Price *et al.* 2010; Surovtseva *et al.* 2009). CST also has nontelomeric roles, and was originally purified from human cells as “DNA Polymerase α accessory factor” (Goulian *et al.* 1990).

Defective telomeres engage nucleases, helicases, and kinases that “repair” the damage and stimulate cell cycle arrest while repair occurs. RPA, the major eukaryotic single-stranded DNA binding protein is, like CST, a heterotrimer, and plays critical roles in DNA repair and the DNA damage checkpoint pathway (Sugitani and Chazin 2015). Interestingly, RPA, also functions at telomeres; for example, RPA binds telomeric ssDNA and promotes telomerase activity (Luciano *et al.* 2012). One view of the relationship between RPA and CST is that RPA binds ssDNA throughout the genome, including at telomeres, whereas CST more specifically binds telomeric ssDNA (Gao *et al.* 2007). But much remains to be learnt about how CST and RPA function at telomeres, and elsewhere in the genome. For example, there is evidence that different components of the CST complex perform different functions (Holstein *et al.* 2014; Lue *et al.* 2014; Lee *et al.* 2016).

Inactivation of Cdc13 using *cdc13-1*—a temperature-sensitive allele—results in extensive 5′–3′ telomeric DNA resection by two nuclease activities, Exo1 and Dna2-Sgs1 (Ngo and Lydall 2010; Ngo *et al.* 2014). The ssDNA generated, which extends to single copy, subtelomeric loci, stimulates the DNA damage checkpoint kinase cascade, which phosphorylates many downstream targets to facilitate cell cycle arrest and DNA repair. The checkpoint response in *cdc13-1* strains is dependent on checkpoint sensors (the 9-1-1 complex, Ddc1, Mec3, and Rad17 in budding yeast), an adaptor (Rad9), a central kinase (Mec1), and effector kinases (Rad53 and Chk1). Checkpoint proteins influence resection, as well as cell cycle arrest, notably the 9-1-1 complex stimulates resection, while Rad9 and Rad53 inhibit resection (Jia *et al.* 2004; Zubko *et al.* 2004; Morin *et al.* 2008).

Many gene deletions suppress or enhance a *cdc13-1* induced growth defect. Broadly, gene deletions that affect normal telomere maintenance enhance *cdc13-1* growth defects, while deletions that disable checkpoint responses suppress *cdc13-1* growth defects (Lydall 2009). Suppressor and enhancer interactions have been measured genome-wide in cells expected to contain telomere defects (*cdc13-1* and *yku70*∆), or more general DNA replication defects (defective in Pol α, Pol δ, and Pol ε) (Addinall *et al.* 2011; Dubarry *et al.* 2015). These genome-wide experiments confirm a pattern seen in earlier experiments, that checkpoint pathways inhibit growth of cells with telomere defects, but improve growth of cells with general replication defects (Weinert *et al.* 1994). This pattern can be rationalized by the fact that telomeric DNA is comparatively unimportant in comparison with the rest of the genome.

To better understand the network that responds to telomere defects, additional mutations, expected to cause telomere, or more general, chromosome damage have been combined with genome-wide libraries of mutations. We then used quantitative fitness analysis (QFA) to measure fitness of these strains at temperatures that induced chronic low-level defects or more acute defects. We assessed fitness of strains with defects in Cdc13, Stn1, and Rfa3. In addition, fitness of *cdc13-1 exo1∆* and *cdc13-1 rad9∆* strains was measured. The measurements illustrate the complexity of the networks that respond to telomere defects. Each genetic defect was affected differently by other second site mutations. Among many interactions, the screens identified a new role for Chk1 in the response to uncapped telomeres.

## Materials and Methods

### Strains

All experiments were performed in W303 or S288C background strains (Table 1).

**Table 1:** Strains used in this study

Strain	Genetic Background	Genotype	Related Figures
DLY640	W303	*MATa*	Figure 4, and Figure S1 and Figure S3 in File S1
DLY1195	W303	*MATα cdc13-1*	Figure 4
DLY1255	W303	*MATa rad9::HIS3 cdc13-1*	Figure S2 in File S1
DLY1256	W303	*MATα rad9::HIS3 cdc13-1*	Figure S2 in File S1
DLY1273	W303	*MATa exo1::LEU2*	Figure 5
DLY1296	W303	*MATa exo1::LEU2 cdc13-1*	Figure 4
DLY1543	W303	*MATa tel1::TRP1 cdc13-1*	Figure S3 in File S1
DLY1544	W303	*MATα tel1::TRP1 cdc13-1*	Figure S3 in File S1
DLY1585	W303	*MATα rad9::KANMX*	Figure S1 in File S1
DLY2234	W303	*MATa rad9::LEU2*	S1 and S2 in File S1
DLY2787	W303	*MATα yku70::LEU2*	Figure 4
DLY2988	W303	*MATa cdc13-1 rad53::HIS3 sml1::KANMX*	Figure 5
DLY3001	W303	*MATα*	Figure S1 and Figure S2 in File S1
DLY4528	W303	*MATa nmd2::HIS3*	Figure S1 in File S1
DLY4557	W303	*MATa cdc13-1 int*	Figure 5
DLY4625	W303	*MATa cdc13-1 int nmd2::HIS3*	Figure 4
DLY4647	W303	*MATa cdc13-1 rad9::HIS3*	Figure 5 and Figure S3 in File S1
DLY4921	W303	*MATa cdc13-1 int exo1::LEU2*	Figure 5
DLY4922	W303	*MATa cdc13-1 rad9::HIS3 exo1::LEU2*	Figure 5
DLY4931	W303	*MATα cdc13-1 rad24::TRP1*	Figure 5
DLY5007	W303	*MATα yku70::LEU2 nmd2::HIS3*	Figure 4
DLY5097	W303	*MATa cdc13-1 exo1::LEU2 rad24::TRP1*	Figure 5
DLY5255	W303	*MATα rad9::LEU2 nmd2::HIS3 cdc13-1 int*	Figure 4
DLY5260	W303	*MATa cdc13-1 cdc15-2 bar1::hisG*	Figure 6
DLY5261	W303	*MATa cdc13-1 cdc15-2 bar1::hisG*	Figure 6
DLY5266	W303	*MATa cdc13-1 cdc15-2 bar1::hisG exo1::LEU2*	Figure 6
DLY5386	S288C	*MATα LEU2::cdc13-1 int::HPHMX rad9::NATMX lyp1∆ can1::MFA1pr-HIS3 ura3 leu2 his3 LYS2+*	Figure 2 and Figure S4 in File S1
DLY5688	S288C	*MATα LEU2::cdc13-1 int::HPHMX lyp1∆ can1::MFA1pr-HIS3 ura3 leu2 his3 LYS2+*	Figure 1, Figure 2, and Figure S4 in File S1
DLY6720	S288C	*MATα LEU2::cdc13-1 int::HPHMX exo1::NATMX lyp1∆ can1::STE2pr-his5 ura3 leu2 his3 LYS2+*	Figure 2 and Figure S4 in File S1
DLY7106	W303	*MATa chk1::HIS3*	Figure 5
DLY7108	W303	*MATa exo2::LEU2 chk1::HIS3*	Figure 5
DLY7110	W303	*MATa cdc13-1 chk1::HIS3*	Figure 5
DLY7112	W303	*MATa cdc13-1 exo1::LEU2 chk1::HIS3*	Figure 5
DLY7143	W303	*MATa cdc13-1 cdc15-2 bar1::hisG chk1::HIS3*	Figure 6
DLY7145	W303	*MATa cdc13-1 cdc15-2 bar1::hisG exo1::LEU2 chk1::HIS3*	Figure 6
DLY7146	W303	*MATa cdc13-1 cdc15-2 bar1::hisG exo1::LEU2 chk1::HIS3*	Figure 6
DLY7747	W303	*MATa exo1::LEU2 nmd2::URA3 cdc13-1*	Figure 4
DLY8460	W303	*MATa*	Figure 5
DLY8767	S288C	*MATα LEU2::stn1-13::HPHMX lyp1::NATMX can1::STE2pr-his5 ura3 leu2 his3 met15 LYS2+*	Figure 1
DLY9181	S288C	*MATα LEU2::rfa3-313::HPHMX lyp1::NATMX can1::STE2pr-his5 ura3 leu2 his3 met15 LYS2+*	Figure 1
DLY9326	S288C	*MATα lyp1::HPHMX::LEU2::NATMX can1::STE2pr-his5 ura3 leu2 his3 met15 LYS2+*	Figure 1
DLY9866	W303	*MATa rad9::LEU2 cdc13-1*	Figure 4 and S3 in File S1
DLY11098	W303	*MATα stn1-13*	Figure S1 in File S1
DLY11099	W303	*MATα stn1-13*	Figure S1 in File S1
DLY11100	W303	*MATα stn1-13 rad9::LEU2*	Figure S1 in File S1
DLY11101	W303	*MATa stn1-13 rad9::LEU2*	Figure S1 in File S1
DLY11102	W303	*MATα stn1-13 rad24::TRP1*	Figure S1 in File S1
DLY11103	W303	*MATa stn1-13 rad24::TRP1*	Figure S1 in File S1
DLY11104	W303	*MATa stn1-13 rad17::TRP1*	Figure S1 in File S1
DLY11105	W303	*MATα stn1-13 rad17::TRP1*	Figure S1 in File S1
DLY11215	W303	*MATα nmd2::URA3 stn1-13*	Figure 4
DLY11216	W303	*MATa stn1-13*	Figure 4
DLY11637	W303	*MATa rfa3-313::KANMX*	Figure S2 in File S1
DLY11696	W303	*MATa rfa3-313::KANMX nmd2::HIS3*	Figure S2 in File S1
DLY11697	W303	*MATα rfa3-313::KANMX nmd2::HIS3*	Figure S2 in File S1
DLY11729	W303	*MATα rfa3-313::KANMX rad9::HIS3*	Figure S2 in File S1
DLY11730	W303	*MATα rfa3-313::KANMX rad9::HIS3*	Figure S2 in File S1
DLY11182	W303	*Matα rad9::HIS3 tel1::TRP1 cdc13-1*	Figure S3 in File S1
DLY11183	W303	*Mata rad9::HIS3 tel1::TRP1 cdc13-1*	Figure S3 in File S1

### QFA

Query strains used are described in Table 2. SGA (synthetic genetic array) was performed as previously described, crossing *cdc13-1*, *cdc13-1 rad9∆*, *cdc13-1 exo1∆*, *stn1-13*, *rfa3-313*, *lyp1∆*, and *ura3∆* with the genome-wide single gene deletion knock-out collection (Tong and Boone 2006; Tong *et al.* 2001). *cdc13-1*, *stn1-13*, and *rfa3-313* were flanked by the selectable HphMX and *LEU2* markers. Each strain also contains a third selectable marker, NATMX. *stn1-13* and *rfa3-313* query strains contained NATMX integrated at the *LYP1* locus. In *cdc13-1 rad9∆* and *cdc13-1 exo1∆* query strains, *RAD9* and *EXO1* were replaced by NATMX.

**Table 2:** QFA screens

Screen No.	Query Strain	Spotting	Media	Temperature
QFA0141	*ura3*	Dilute	SDM_rhk_CTGN	27°, UD_X3
QFA0132	*lyp1*	Concentrated	SDM_rhlk_CTGNH	30°, 33°
QFA0140	*cdc13-1*	Dilute	SDM_rhlk_CTGH	27°, UD_X3
QFA0142	*rad9Δ cdc13-1*	Dilute	SDM_rhlk_CTGNH	27°, UD_X1
QFA0051	*exo1Δ cdc13-1*	Dilute	SDM_rhlk_CTGNH	27°, 30°
QFA0136	*stn1-13*	Concentrated	SDM_rhlk_CTGNH	33°
QFA0131	*rfa3-313*	Concentrated	SDM_rhlk_CTGNH	30°

For QFA, strains were inoculated into 200 µl liquid medium in 96-well plates, and grown for 2 d at 20° without shaking, as previously described (Dubarry *et al.* 2015). After resuspension, saturated cultures were spotted onto solid agar plates, either directly, or after diluting in water, and agar plates were incubated and imaged as before (Addinall *et al.* 2011; Dubarry *et al.* 2015). For *ura3∆* up-down (UD) and the *cdc13-1* (UD) assays, plates were incubated at 36°, for 5 hr, followed by 20° for 5 hr, three times, then plates were kept at 20° for the remaining time. For the *rad9∆ cdc13-1* (UD) assay, plates were incubated at 36° for 8 hr, followed by incubation at 23° for the remaining time.

### Small-scale spot tests

To examine colony morphology, size, and heterogeneity, yeast strains were struck for single colonies. To measure a strain’s fitness phenotype by spot test, several colonies were pooled and inoculated into 2 ml YEPD, and incubated on a wheel at 23° overnight until saturation. Fivefold serial dilutions of saturated cultures were spotted onto agar plates using a 48- or 96-prong replica plating device. Plates were incubated at different temperatures for 2–3 d before being photographed.

### Cell cycle analysis

W303 strains containing *cdc13-1 cdc15-2 bar1∆* mutations were grown at 23°, and arrested in G1 using α-factor. Strains were then released from G1 at 36° to induce telomere uncapping. Samples were taken periodically, and cell cycle position was determined using DAPI staining (Zubko *et al.* 2004).

### Quantitative amplification of single-stranded DNA

ssDNA levels were determined using quantitative amplification of single-stranded DNA (QAOS), as previously described (Holstein and Lydall 2012).

### Profilyzer and DIXY

Profilyzer and DIXY are web-based tools for visualizing and comparing results from multiple QFA screens at once (Dubarry *et al.* 2015). Profilyzer consists of various custom-built R functions inside a Shiny framework (Chang *et al.* 2015). A live instance of Profilyzer for this manuscript can be found at: http://research.ncl.ac.uk/qfa/Holstein2017. The DIXY instance for this manuscript can be accessed at: http://bsu-srv.ncl.ac.uk/dixy-telo. Data and source code underlying these instances, can be found on GitHub: https://github.com/lwlss/Holstein2016.

### Data availability

Tab-delimited text files containing the raw fitness measurements and estimates of genetic interaction strengths underlying figures from this article can be found in the following GitHub repository: https://github.com/lwlss/Holstein2016. All yeast strains listed in Table 1 are available on request.

## Results

Previous comparisons between genome-wide genetic interaction screens of *cdc13-1* and *yku70∆* strains revealed similarities and differences in the types of interactions observed (Addinall *et al.* 2011). For example, the *exo1∆* mutation suppresses *cdc13-1* and *yku70∆* induced growth defects, while *nmd∆* mutations suppress *cdc13-1* but enhance *yku70∆* growth defects. Therefore, this genome-wide genetic interaction approach was extended to examine interactions with new mutations.

### STN1

We first examined genetic interactions affecting fitness of *stn1-13* mutants. Stn1, like Cdc13, is an essential component of the CST complex (Cdc13-Stn1-Ten1) that binds telomeric ssDNA and affects DNA replication. There is also evidence that Cdc13 and Stn1 perform different functions. For example, a *stn1-186t* truncated allele is synthetically lethal with the *rad9∆* mutation (Petreaca *et al.* 2007), whereas, in contrast, the *cdc13-1* mutation is suppressed by *rad9∆* (Zubko *et al.* 2004). Similarly, mutations that completely bypass the requirement for *CDC13*, and permit *cdc13∆* cells to grow, do not bypass the requirement for *STN1* (Holstein *et al.* 2014). If Stn1 and Cdc13 perform different functions then these functions might be revealed by specific genetic interactions.

A temperature sensitive *stn1-13* ts allele was crossed to a genome-wide collection of mutations (*yfg∆*), and fitness of the resulting double mutants was measured by QFA. *stn1-13* has a higher permissive temperature than *cdc13-1*, and, therefore, double mutants were cultured at 33°, a temperature that moderately inhibits growth of *stn1-13* strains. The overall pattern of genetic interactions observed in *stn1-13* cells is different to that previously reported for *cdc13-1* cells, with a tighter clustering of fitness measurements (Figure 1, B and C). The different patterns could be due to different functions of Cdc13 and Stn1, the different properties of the two alleles, or technical differences between the genome-wide experiments (see Table 2), which were performed more than five years apart.

To help assess the technical quality of the *stn1-13* experiment, we highlight the positions of 19 diagnostic gene deletions that play roles in telomere physiology, or in telomere-defective strains (Table 3). In particular, among these 19, are five sets of gene deletions affecting the checkpoint sliding clamp, nonsense-mediated mRNA decay (NMD), the Ku complex, the MRX complex, or telomerase (Figure 1A). In principle, if members of a protein complex always function together, then each individual deletion should show similar genetic interactions to other deletions affecting the same complex. Reassuringly, individual deletions affecting all five complexes were similarly colocated. In particular, mutations affecting the checkpoint clamp/loader, or NMD, caused similar increases in fitness of *stn1-13* strains (located near the top of Figure 1B). On this basis, we conclude that the *stn1-13* genome-wide experiment reports meaningful genetic interactions.

**Table 3:** List of proteins affected by gene deletions highlighted in QFA screens

Standard Name	Complex	Description from *Saccharomyces* Genome Database
Ddc1	9-1-1 sliding clamp	DNA damage checkpoint protein; part of a PCNA-like complex required for DDR, required for pachytene checkpoint to inhibit cell cycle in response to unrepaired recombination intermediates; potential Cdc28p substrate; forms nuclear foci upon DNA replication stress
Rad17	9-1-1 sliding clamp	Checkpoint protein; involved in the activation of the DNA damage and meiotic pachytene checkpoints; with Mec3p and Ddc1p, forms a clamp that is loaded onto partial duplex DNA; homolog of human and *Schizosaccharomyces pombe* Rad1 and *Ustilago maydis* Rec1 proteins
Rad24	9-1-1 sliding clamp	Checkpoint protein; involved in the activation of the DNA damage and meiotic pachytene checkpoints; subunit of a clamp loader that loads Rad17p-Mec3p-Ddc1p onto DNA; homolog of human and *S. pombe* Rad17 protein
Mre11	MRX complex	Nuclease subunit of the MRX complex with Rad50p and Xrs2p; complex functions in repair of DNA DSBs and in telomere stability; Mre11p associates with Ser/Thr-rich ORFs in premeiotic phase; nuclease activity required for MRX function; widely conserved; forms nuclear foci upon DNA replication stress
Rad50	MRX complex	Subunit of MRX complex with Mre11p and Xrs2p; complex is involved in processing DNA DSBs in vegetative cells, initiation of meiotic DSBs, telomere maintenance, and nonhomologous end joining; forms nuclear foci upon DNA replication stress
Xrs2	MRX complex	Protein required for DNA repair; component of the Mre11 complex, which is involved in DSBs, meiotic recombination, telomere maintenance, and checkpoint signaling
Nam7	NMD	ATP-dependent RNA helicase of the SFI superfamily; involved in NMD; required for efficient translation termination at nonsense codons and targeting of NMD substrates to P-bodies; binds to the small ribosomal subunit via an interaction with Rps26; forms cytoplasmic foci upon DNA replication stress
Nmd2	NMD	Protein involved in the NMD pathway; interacts with Nam7p and Upf3p; involved in telomere maintenance
Upf3	NMD	Component of the NMD pathway; along with Nam7p and Nmd2p; involved in decay of mRNA containing nonsense codons; involved in telomere maintenance
Yku70	Ku heterodimer	Subunit of the telomeric Ku complex (Yku70p-Yku80p); involved in telomere length maintenance, structure, and telomere position effect; required for localization of telomerase ribonucleoprotein to nucleus via interaction with the TLC1 guide RNA; relocates to sites of double-strand cleavage to promote nonhomologous end joining during DSB repair
Yku80	Ku heterodimer	Subunit of the telomeric Ku complex (Yku70p-Yku80p); involved in telomere length maintenance, structure, and telomere position effect; required for localization of telomerase ribonucleoprotein via interaction with the TLC1 guide RNA; relocates to sites of double-strand cleavage to promote nonhomologous end joining during DSB repair
Est1	Telomerase	TLC1 RNA-associated factor involved in telomere length regulation; recruitment subunit of telomerase; has G-quadruplex promoting activity required for telomere elongation; possible role in activating telomere-bound Est2p-TLC1-RNA; EST1 has a paralog, EBS1, that arose from the whole genome duplication
Est2	Telomerase	Reverse transcriptase subunit of the telomerase holoenzyme; essential for telomerase core catalytic activity, involved in other aspects of telomerase assembly and function; mutations in human homolog are associated with aplastic anemia.
Est3	Telomerase	Component of the telomerase holoenzyme; involved in telomere replication
Rif1	Rap1 interacting factor	Protein that binds to the Rap1p C-terminus; acts synergistically with Rif2p to help control telomere length and establish telomeric silencing; involved in control of DNA replication; contributes to resection of DNA DSBs; deletion results in telomere elongation
Rif2	Rap1 interacting factor	Protein that binds to the Rap1p C-terminus; acts synergistically with Rif1p to help control telomere length and establish telomeric silencing; deletion results in telomere elongation; RIF2 has a paralog, ORC4, that arose from the whole genome duplication
Rad9		DNA damage-dependent checkpoint protein; required for cell-cycle arrest in G1/S, intra-S, and G2/M, plays a role in postreplication repair (PRR) pathway; transmits checkpoint signal by activating Rad53p and Chk1p; hyperphosphorylated by Mec1p and Tel1p; multiple cyclin dependent kinase consensus sites, and the C-terminal BRCT domain contribute to DNA damage checkpoint activation; Rad9p Chk1 Activating Domain (CAD) is phosphorylated at multiple sites by Cdc28p/Clb2p
Chk1		Serine/threonine kinase and DNA damage checkpoint effector; mediates cell cycle arrest via phosphorylation of Pds1p; phosphorylated by checkpoint signal transducer Mec1p; homolog of *S. pombe* and mammalian Chk1 checkpoint kinase
Tel1		Protein kinase primarily involved in telomere length regulation; contributes to cell cycle checkpoint control in response to DNA damage; acts with Red1p and Mec1p to promote interhomolog recombination by phosphorylation of Hop1; functionally redundant with Mec1p; regulates P-body formation induced by replication stress; homolog of human ataxia-telangiectasia mutated (ATM) gene
Exo1		5′–3′ exonuclease and flap-endonuclease; involved in recombination, DSB repair, MMS2 error-free branch of the PRR pathway and DNA mismatch repair; role in telomere maintenance; member of the Rad2p nuclease family, with conserved N and I nuclease domains; relative distribution to the nucleus increases upon DNA replication stress; EXO1 has a paralog, DIN7, that arose from the whole genome duplication

It is possible to speculate on the molecular basis for some of these genetic interactions. For example, disabling the NMD pathway leads to overexpression of the CST components, Ten1 and Stn1 (Dahlseid *et al.* 2003; Addinall *et al.* 2011), and an increase in Stn1 or Ten1 levels could readily suppress *stn1-13* and *cdc13-1* mutations (Figure 1, B and C). The suppressive effects of mutations affecting the checkpoint clamp/loader are most likely because telomere defects in *stn1-13* cells stimulate the DNA damage checkpoint pathway to inhibit cell growth. Curiously, inactivation of the Ku complex (*yku70∆* and *yku80∆*), or telomerase (*est1∆* and *est3∆*) resulted in comparatively minor reduction of the fitness of *stn1-13* cells, in comparison with their effects on *cdc13-1* strain fitness (Figure 1, B and C). One interpretation of the difference is that *stn1-13* cells contain more defects than *cdc13-1* cells at nontelomeric loci.

Reassuringly, the *stn1-13* high-throughput screen reproduced the observation that fitness defects caused by *stn1* mutations are enhanced by *rad9∆* (Figure 1B) (Petreaca *et al.* 2007). It is likely informative that *rad9∆* enhances, whereas *rad17∆*, *rad24∆*, and *ddc1∆* mildly suppress, *stn1-13* fitness defects (Figure 1B). In contrast, *rad9∆*, *rad17∆*, *rad24∆*, and *ddc1∆* each suppress *cdc13-1* (Figure 1C). We confirmed that *rad9∆* enhances while *rad17*∆ and *rad24*∆ mildly suppress *stn1-13* in the different, W303, genetic background (Figure S1 in File S1). These genetic interactions suggest a different function for Rad9 in *stn1-13* and *cdc13-1* cells. Furthermore, previous experiments showed that *rad9∆ and rad24∆* mutations were synthetically lethal with a truncated *stn1-186t* allele, suggesting that *RAD9/RAD24*-dependent checkpoint function is essential in *stn1-186t* cells (Petreaca *et al.* 2007). In this respect, *stn1-186t* appears to be a mutation causing general DNA replication defects, rather than telomere defects, because it interacts more similarly to mutations affecting Pol α, Pol δ, or Pol ε, than to *cdc13-1* (Addinall *et al.* 2011; Dubarry *et al.* 2015). Overall, it is clear that there are similarities and differences in the genetic interactions observed in *cdc13-1*, *stn1-13*, and *stn1-186t* strains, presumably reflecting the fact that each mutation causes similar, but distinct, molecular defects.

### RFA3

The heterotrimeric Replication Protein A (RPA), consisting of Rfa1, Rfa2, and Rfa3, binds ssDNA, and plays critical roles in DNA replication and the DNA damage response. To explore the functional relationship between CST and RPA, genetic interactions were measured in strains defective in the small subunit of RPA containing the temperature sensitive *rfa3-313* allele (Figure 1D).

It is clear that deletions of members of the NMD complex (*nam7∆*, *nmd2∆*, and *upf3∆*) caused a decrease in fitness in *rfa3-313* mutants (Figure 1D), the opposite effect to that observed in *stn1-13* and *cdc13-1* mutants (Figure 1, B and C). Checkpoint mutations (*chk1∆*, *rad9∆*, *rad17∆*, *rad24∆*, and *ddc1∆*) slightly enhanced fitness defects, or were comparatively neutral, in *rfa3-313* mutants. We confirmed that *nmd∆* mutations enhance, whereas the *rad9*∆ checkpoint mutation is comparatively neutral in W303, *rfa3-313* cells (Figure S2 in File S1). *nmd∆* mutations increase levels of Stn1 and Ten1 (Dahlseid *et al.* 2003), the CST subunits equivalent to RPA subunits Rfa2 and Rfa3. Therefore, it is plausible that increased levels of Stn1/Ten1 exacerbate fitness defects caused by the *rfa3-313* allele, causing a dose-dependent dominant-negative interaction. Overall, it is noteworthy that the pattern of genetic interactions observed in *rfa3-313* strains, presumably with more global chromosome defects, is markedly different to that seen in *cdc13-1* or *stn1-13* strains, which presumably have more telomere-specific defects.

### The effects of Rad9 and Exo1 on the response to cdc13-1 defects

A large network of proteins coordinates the response of cells to damaged telomeres. Deletion of *RAD9*, a checkpoint gene, or *EXO1*, a nuclease gene, similarly improve the fitness of *cdc13-1* strains grown at semipermissive temperature (Figure 1C). However, Rad9 and Exo1 contribute in very distinct ways to fitness of *cdc13-1* mutants (Zubko *et al.* 2004). Rad9 is critical for the cell cycle arrest pathway that responds to *cdc13-1* defects and binds chromatin to inhibit nucleases that generate ssDNA at defective telomeres. Exo1 is one of the nucleases that generate ssDNA in *cdc13-1* and other telomere defective strains. Therefore, we screened the genome-wide knock-out library for genes interacting with *exo1∆* or *rad9∆* in a *cdc13-1* background, aiming to better define the structure of the DDR network that is active in *cdc13-1* cells.

**Figure 2:**
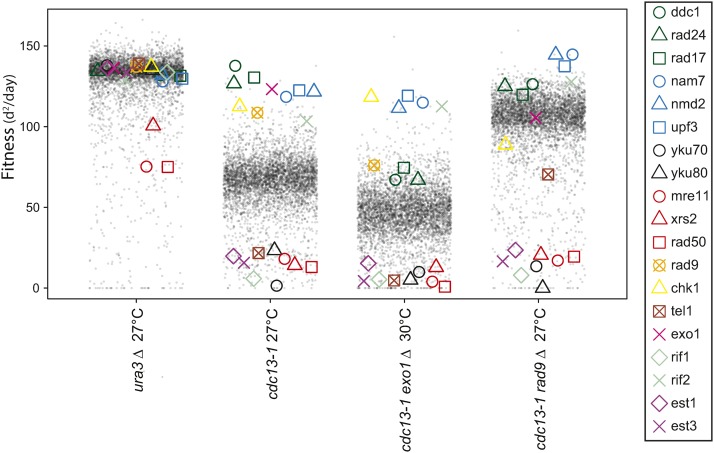
Effects of *rad9∆* or *exo1∆* on the fitness of *cdc13-1* strains. Fitness profile showing the effects of ∼5000 *yfg∆* library mutations on the fitness of *ura3∆*, *cdc13-1*, *cdc13-1 rad9∆*, and *cdc13-1 exo1∆* strains. Each point represents the fitness of one gene deletion strain in each combination of genetic background and temperature. Fitness is measured as in Figure 1. Nineteen telomere-related genes from Figure 1A are highlighted with colored symbols.

Figure 2 allows us to compare the effects of gene deletions in cells with the *cdc13-1* mutation, with or without *exo1*∆ or *rad9*∆ mutations. *cdc13-1 rad9∆* strains are checkpoint defective, but nuclease hyperactive, while *cdc13-1 exo1∆* cells are checkpoint proficient, but nuclease hypoactive. The general effect of *rad9*∆ on the library of *cdc13-1 yfg∆* mutants was to improve fitness, as seen by the increased fitness of most library strains. The global effects of *exo1*∆ are harder to discern, because the fitness of *cdc13-1 exo1*∆ strains was measured at 30° (a higher temperature than 27°, to better assess the effects of the telomere defect). It is interesting to compare the 19 gene deletions from Figure 1A in the different contexts. For example, deletions affecting the 9-1-1 complex (*rad17∆*, *rad24∆*, and *ddc1∆*), or NMD (*nam7∆*, *nmd2∆*, and *upf3∆*), are similarly strong suppressors of *cdc13-1*. But in *cdc13-1 rad9∆* and *cdc13-1 exo1∆* strains, *nmd*∆ mutations are clearly fitter than *911*∆ checkpoint mutations. It is also notable that *chk1∆* had a stronger suppressive effect than other checkpoint gene deletions in *cdc13-1 exo1∆* strains, and this is investigated further in Figure 6.

Perhaps as expected, numerous deletions affecting telomerase, the Ku complex, the MRX complex and Rif1, known to play important roles in telomere function, strongly reduced fitness in *cdc13-1*, *cdc13-1 rad9∆*, and *cdc13-1 exo1∆* strains (Figure 2). Interestingly, *tel1∆* showed a different pattern, it reduced fitness in *cdc13-1* and *cdc13-1 exo1∆* strains, but less so in the *cdc13-1 rad9∆* context (right column Figure 2). Whether these differences are due to the absence of a functional checkpoint pathway in *cdc13-1 rad9∆* cells, or other reasons, is unclear. Nevertheless, we confirmed the effects of *tel1*∆ and *rad9∆* in *cdc13-1* strains in small-scale W303 spot tests (Figure S3 in File S1).

### Acute telomere defects

Culturing *cdc13-1* cells at semipermissive temperatures (*e.g.*, 27°) allows assessment of the effects of genes on fitness of cells with chronic, low-level, defects. In this assay *RAD9* and *EXO1* have very similar effects (Figure 1C). A complementary approach is to identify genes that affect the viability of *cdc13-1* mutants after acute, high-level damage (Addinall *et al.* 2008). After acute damage, *RAD9* and *EXO1* have opposite effects. Exo1 reduces, while Rad9 protects, viability of *cdc13-1* cells (Zubko *et al.* 2004). The different effects of *RAD9*, *EXO1*, and other genes can be explained by their effects on ssDNA accumulation at uncapped telomeres, with Exo1 stimulating ssDNA production and Rad9 inhibiting production (Zubko *et al.* 2004; Jia *et al.* 2004).

**Figure 3:**
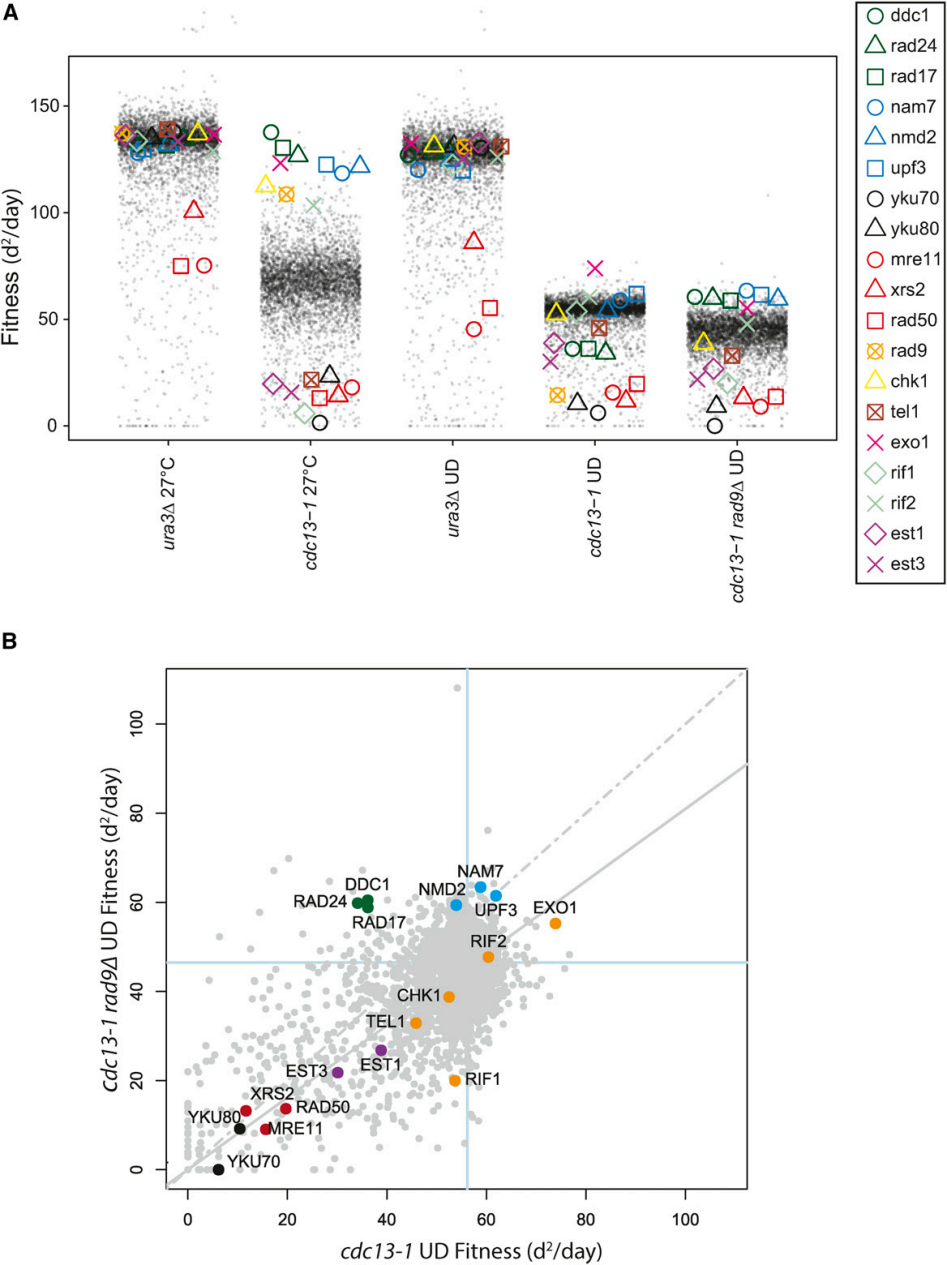
The effects of a library of *yfg∆* mutations on fitness of cells after exposure to chronic or acute telomere defects. (A) Fitness profile comparing the effects of ∼5000 *yfg∆* library mutations on the fitness of strains indicated after chronic (27°) or acute (UD) exposure to telomere defects. In UD experiments, cells were exposed to short periods of incubation at 36° (see *Materials and Methods*). Data are plotted as in Figure 2. (B) Fitness plot comparing evidence for genetic interactions between *rad9∆* and ∼5000 *yfg∆* deletions in a *cdc13-1* background after acute telomere uncapping. Data are plotted as in Figure 1.

To identify genes that affect cell fitness after acute exposure to telomere defects, we performed genome-wide experiments, in which cells were exposed to acute periods of incubation at 36° followed by recovery at 23°. We call this type of temperature cycling protocol an up-down (UD) assay. Importantly, the previously reported opposing effects of *rad9∆* and *exo1∆* on viability of *cdc13-1* cells in UD assays were confirmed in the genome-wide experiments, with *exo1∆* strains being among the most fit, and *rad9*∆ strains being among the least fit (Figure 3A) (Zubko *et al.* 2004; Addinall *et al.* 2008). The genome-wide experiments also confirmed that *rad9∆* strains were less viable than *rad17∆*, *ddc1∆*, and *rad24∆* strains in the *cdc13-1* (UD) context, consistent with what has been reported for *rad24∆* (Figure 3A) (Zubko *et al.* 2004).

It is informative to compare genetic interactions observed in *cdc13-1* (UD) and *cdc13-1 rad9∆* (UD) screens, since the *rad9∆* mutation strongly sensitizes *cdc13-1* strains to acute high temperature. Perhaps the most notable difference is in the effects of mutations affecting the 9-1-1 complex; *rad17∆*, *ddc1∆*, and *rad24∆* are among the least fit strains in the *cdc13-1* (UD) context, but among the most fit in the *cdc13-1 rad9∆* (UD) context (Figure 3A). Figure 3B directly compares fitness in the two *cdc13-1* UD contexts, and confirms that mutations affecting the 9-1-1 complex are outliers. In contrast, most gene deletions of the 19 from Figure 1A lie along the regression line in Figure 3B. The differential effects of the 9-1-1 complex in the two situations can be explained by the fact that 9-1-1 has two important functions in *cdc13-1* strains. The 9-1-1 complex is critical for protecting cell viability of *cdc13-1* strains because it is necessary for checkpoint arrest. On the other hand, in *cdc13-1 rad9∆* strains, where there is no cell cycle arrest, 9-1-1 contributes to cell death by facilitating nuclease activities (Ngo and Lydall 2015; Zubko *et al.* 2004).

That most gene deletions have similar effects on fitness of *cdc13-1* and *cdc13-1 rad9∆* strains after UD assays suggests that there is comparatively little difference between the structure of telomeres in the two contexts. For example, suppressor mutations, like *exo1∆*, or enhancers, like *yku70∆* and *yku80∆*, seem to be similarly important in both contexts. On the other hand, in addition to those affecting 9-1-1, there were other gene deletions that behaved differently in *cdc13-1 rad9∆* *vs.* *cdc13-1* strains: *rif1∆* strongly enhanced, and *nam7∆*, *nmd2∆*, and *upf3∆* strongly suppressed, in the *rad9∆ cdc13-1* (UD) context. In summary, there are informative similarities and differences in the effects of gene deletions of the viability of *cdc13-1* and *cdc13-1 rad9∆* strains after acute exposure to telomere defects.

In UD assays, *yku70∆* and *yku80∆* mutations, affecting the Ku heterodimer, reduced the fitness of *cdc13-1* and *cdc13-1 rad9*∆ cells more than *est1∆* and *est3∆* mutations, affecting telomerase (Figure 3A). This pattern contrasts to what was seen at 27°, after chronic low level *cdc13-1* telomere damage, when the effects of *yku70∆*, *yku80∆*, *est1∆*, and *est3∆* were all similar to each other. The effects of the Ku heterodimer in *cdc13-1* strains cultured at 36° may be because the Ku heterodimer protects telomeres from the Exo1 nuclease, particularly at high temperature (Maringele and Lydall 2002), and because Ku and Cdc13 function redundantly to cap the telomere (Polotnianka *et al.* 1998).

### Effects of different gene deletions across several telomere defective strains

The data in Figure 1, Figure 2, and Figure 3 shows thousands of genetic interactions that are potentially informative about telomere and chromosome biology. Out of necessity, only a tiny fraction of these interactions have been highlighted. Therefore, to allow others to explore these data, to identify other potentially informative genetic interactions, the data are available via two interactive web tools (Dubarry *et al.* 2015). One of these, DIXY (Dynamic Interactive XY plots), shows fitness data in a format similar to Figure 1 (http://bsu-srv.ncl.ac.uk/dixy-telo/). DIXY also allows generation of scatter plots, and for any gene, or genes, to be highlighted across plots. For example, Figure S4 in File S1 shows a number of pairwise comparisons of the data in Figure 2.

**Figure 4:**
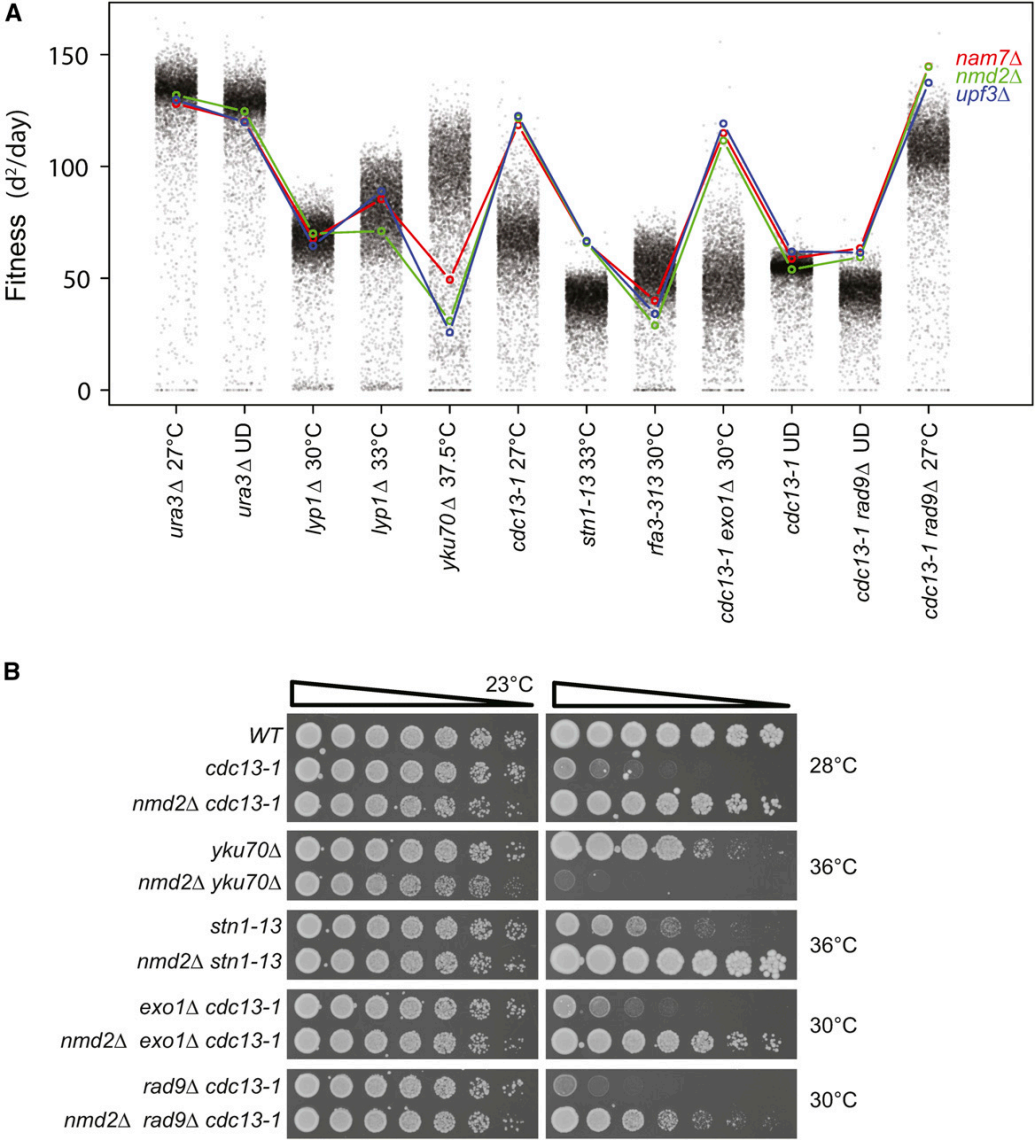
Effects of gene deletions affecting the NMD pathway across a range of telomere defective backgrounds. (A) Profilyzer fitness profiles comparing the effects of *nam7∆*, *nmd2∆*, and *upf3∆* mutations on fitness across all the genome-wide screens presented in Figure 1, Figure 2, and Figure 3. (B) Saturated cultures of the yeast strains indicated (see Table 1) were fivefold serially diluted in water, spotted onto YEPD agar plates, and incubated at the indicated temperatures for 2 d before being photographed.

A second tool, Profilyzer (http://research.ncl.ac.uk/qfa/Holstein2016), shows the effects of mutations across more than two screens, and generates interactive plots similar to Figure 2. Figure 4A illustrates use of Profilyzer to show fitness profiles of three gene deletions affecting NMD (*nam7∆*, *nmd2∆*, and *upf3∆* mutations) across 12 independent, but related, genome-wide screens. The *nmd∆* mutations had minor effects on fitness of control strains (*ura3∆* and *lyp1∆*), increased fitness of *cdc13-1*, *stn1-13*, *cdc13-1 exo1∆*, and *cdc13-1 rad9∆* strains, and *rad9∆ cdc13-1* strains after UD treatments. In contrast, the *nmd∆* mutations exacerbated fitness defects of *yku70∆* and *rfa3-313* strains. Figure 4B confirms that the *nmd2∆* mutation recapitulates many of these interactions in the W303 genetic background.

**Figure 5:**
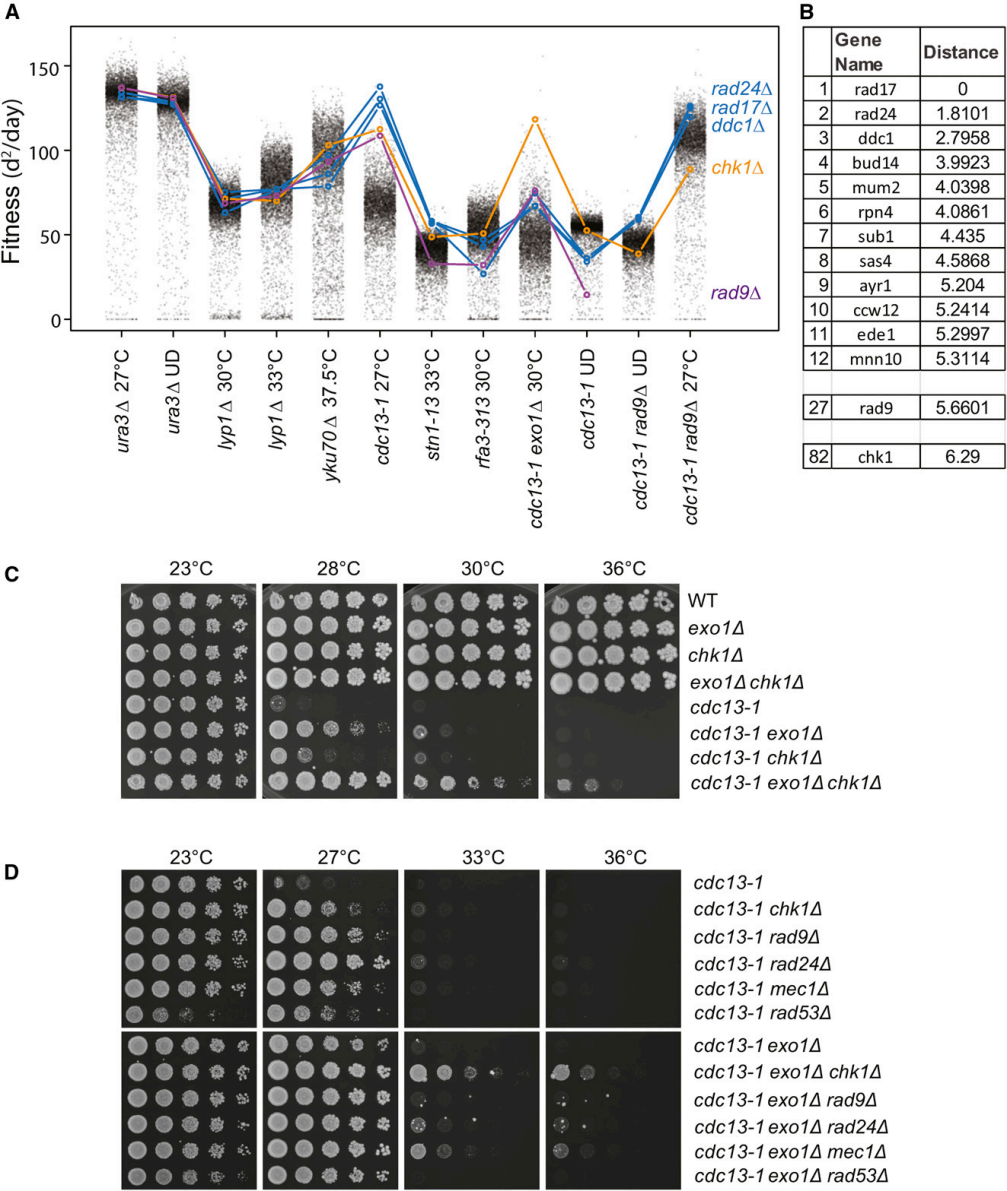
Effects of gene deletions affecting the DNA damage checkpoint pathway across a range of telomere defective backgrounds. (A) Profilyzer fitness profiles comparing the effects of *ddc1∆*, *rad24∆*, *rad17∆*, *rad9∆*, and *chk1∆* mutations on fitness across all the screens presented in Figure 1, Figure 2, and Figure 3. (B) List of gene deletions with most similar fitness profiles to *rad17∆* out of ∼5000 examined, including *rad9*∆ (position 27) and *chk1∆* (position 82). (C, D) Yeast cultures treated as in Figure 4B.

Profilyzer was used to compare the fitness profiles of *chk1∆*, *ddc1∆*, *rad9∆*, *rad17∆*, and *rad24∆*, deletions affecting the checkpoint response. As expected, *ddc1∆*, *rad17∆*, and *rad24∆*, *i.e.*, mutations affecting the 9-1-1 complex, showed the most similar patterns, whereas other checkpoint mutations, *chk1∆* and *rad9∆*, were somewhat different (Figure 5A). For example, *chk1∆* behaved differently to 9-1-1 complex mutations, particularly in the context of *cdc13-1*, *cdc13-1 exo1∆*, or *cdc13-1 rad9∆* mutations (Figure 5A).

Profilyzer also permits identification of gene deletions with fitness profiles most similar to any query gene deletion across some or all screens. To illustrate this, the 11 most similar profiles to *rad17*∆ are shown in Figure 5B. Reassuringly, given their known functions, *rad24∆* and *ddc1∆* had the closest profiles to *rad17∆*, and *rad9∆* and *chk1∆* were among the top 100 most similar profiles, out of 5000 (Figure 5B).

### Chk1 affects ssDNA production

*chk1*∆ had a notably stronger suppressive effect than *ddc1∆*, *rad9∆*, *rad17∆*, and *rad24∆* mutations in *exo1∆ cdc13-1* strains (Figure 5A). Additionally, in the W303 genetic background, *exo1∆* and *chk1∆* double mutations strongly suppressed the temperature sensitivity of *cdc13-1* strains, permitting some growth at 36° (Figure 5C). Consistent with the genome-wide experiments, *chk1∆* was also a stronger suppressor of *cdc13-1 exo1∆* fitness defects in comparison with *rad9∆*, *rad24∆*, *mec1∆*, or *rad53∆* checkpoint mutations, in W303 (Figure 5D). We conclude that *chk1∆* is an unusually strong suppressor of *cdc13-1 exo1∆* growth defects, and this is most likely due to Chk1 having a checkpoint-independent role(s).

**Figure 6:**
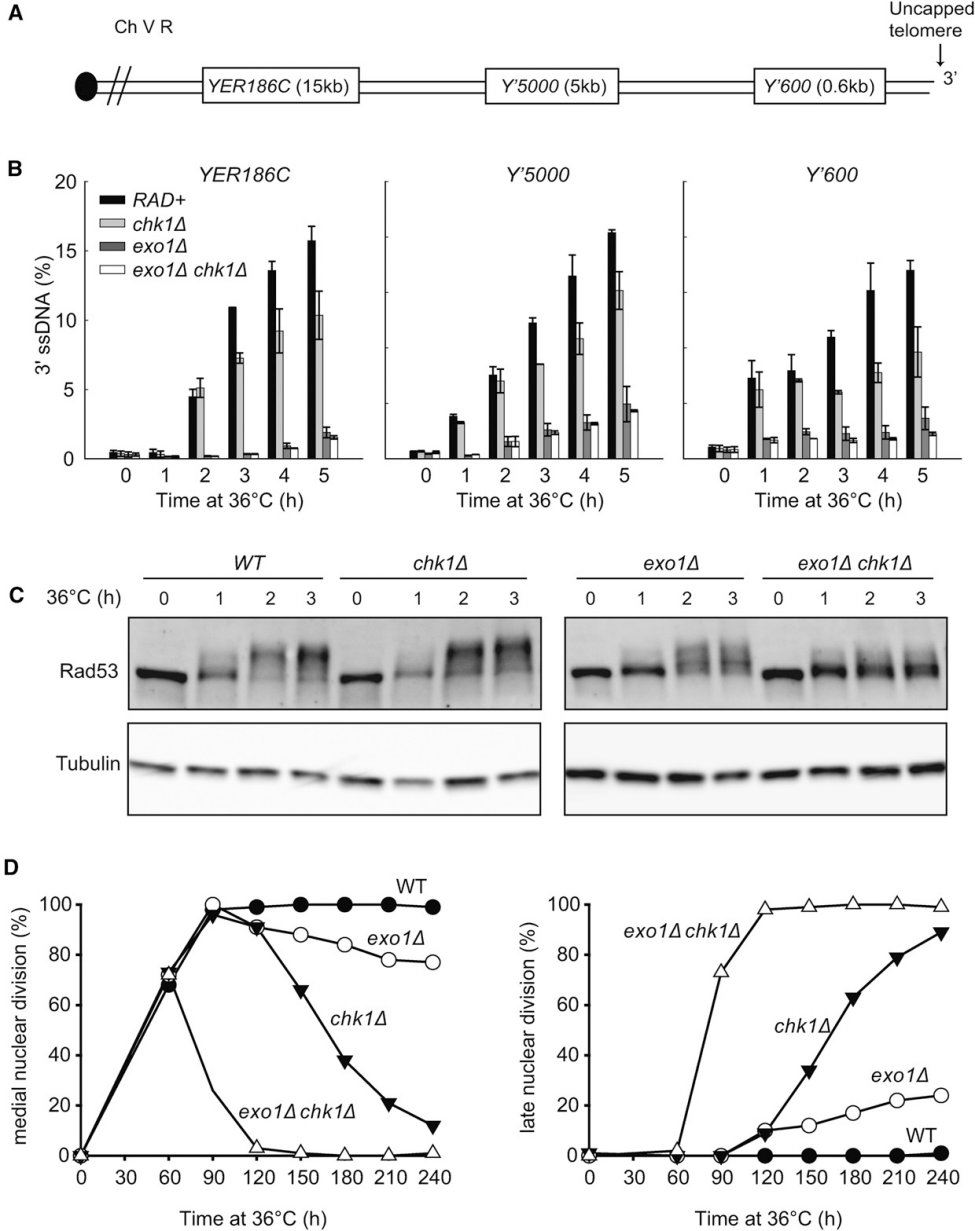
Chk1 stimulates resection, Rad53 phosphorylation and checkpoint activation in response to telomere defects. (A) Map of the right arm of Chromosome V. (B) Quantification of 3′ ssDNA accumulation at loci indicated following telomere uncapping. All strains contain *cdc13-1 cdc15-2 bar1∆* mutations (Table 1). Cells were arrested in G1 with α-factor at 23°, then released at 36° (Lydall and Weinert 1995). ssDNA was measured using QAOS (Booth *et al.* 2001). The data and error bars plotted are the mean and SEM from two independent experiments. (C) Yeast strains with the indicated genotypes (all in *cdc13-1 cdc15-2 bar1∆* background) were subjected to western blot analysis with anti-Rad53 and anti-tubulin antibodies at the times indicated. (D) Cell cycle position of the indicated *cdc13-1 cdc15-2 bar1∆* strains was assessed by counting DAPI-stained cells.

DNA resection is a critically important DNA damage response in *cdc13-1* cells, and, therefore, we hypothesized that Chk1 might stimulate resection. To test this, we examined resection in synchronous cultures of *cdc13-1* strains at high temperature. ssDNA was measured at *Y*’*600* and *Y*’*5000*, located in the Y’ subtelomeric elements, present on two-thirds of budding yeast chromosome ends (including the right telomere of chromosome V, Figure 6A). In addition, ssDNA accumulation at a single copy locus *YER186C*, 15 kb from the right telomere of chromosome V, was measured (Figure 6A). Consistent with previous findings, accumulation of 3′ ssDNA at *Y*’*600* and *Y*’*5000* in wild-type strains was detected after 1 hr, and at *YER186C* after 2 hr (Figure 6B) (Zubko *et al.* 2004). Importantly, lower levels of ssDNA were observed in *chk1∆* mutants at all loci examined, suggesting that Chk1 does indeed stimulate telomere resection. The effect of *chk1∆* was clearly not as strong as *exo1∆*, which helps explain why previous experiments did not report an effect of Chk1 on resection in *cdc13-1* strains (Jia *et al.* 2004). Deleting *CHK1* in *cdc13-1 exo1∆* mutants further reduced ssDNA, especially at Y’600, suggesting that Chk1 may stimulate Sgs1-dependent resection. The small effect of Chk1 on resection is possibly because Sgs1-dependent resection is weak in *cdc13*-1 and *cdc13-1 exo1∆* strains (Ngo *et al.* 2014; Ngo and Lydall 2010).

To search for additional evidence supporting a role of Chk1 in Sgs1-dependent resection, we examined Rad53 phosphorylation—a downstream product of resection. We previously showed that Sgs1 stimulates Rad53 phosphorylation in *cdc13-1 exo1*∆ strains (Ngo and Lydall 2010). Consistent with previous findings, we detected strong Rad53 phosphorylation in *cdc13-1* strains after 2 hr at 36°, and this was slightly reduced in *cdc13-1 exo1*∆ strains (Figure 6C) (Ngo and Lydall 2010). *chk1∆* did not strongly reduce Rad53 phosphorylation in *cdc13-1* strains, but did so in *cdc13-1 exo1∆* strains. Thus, in this Rad53 assay, *chk1∆* mimics *sgs1∆*, suggesting that Chk1 stimulates Sgs1-dependent resection, and thereby Rad53 phosphorylation (Ngo and Lydall 2010).

Previously, it was shown that inactivation of Exo1 and Sgs1-dependent pathways of resection was insufficient to permit *cdc13-1* (*exo1*∆ *sgs*1∆) cells to grow at 36°, because Rad9-dependent cell cycle arrest was still activated (Ngo and Lydall 2010). Therefore, we wondered how *cdc13-1 exo1*∆ *chk1*∆ cells could grow so well at 36° (Figure 5C). We hypothesized that Chk1, like Rad9, is important for cell-cycle arrest of *cdc13-1 exo1*∆ strains. Indeed, when we examined cell-cycle arrest of *cdc13-1 exo1∆ chk1∆* strains, assessing the fraction of cells arrested at medial nuclear division, we saw no evidence for arrest (Figure 6D). In contrast, *cdc13-1* cells remained fully arrested for at least 4 hr, while *exo1∆* or *chk1∆* strains showed mild checkpoint defects, with ∼10% of cells failing to maintain arrest by 2 hr. At later times, *chk1∆* strains showed a more severe checkpoint defect than *exo1∆* strains, such that, by 4 hr, >80% of *chk1∆* cells escaped arrest. We conclude that Chk1 is critical for DNA damage checkpoint activation in *cdc13-1 exo1*∆ cells.

## Discussion

Yeast telomeres resemble mammalian telomeres in many respects, most notably relying on telomerase as a means to overcome the end replication problem. Here, we systematically explored genetic interactions that suppress or enhance different types of genetic defect in budding yeast. Most of the genetic defects examined here are associated with changes to telomeric DNA structure, and, on this basis, we classify them as causing telomere defects. However, the association of particular mutations with telomere defects cannot exclude the possibility that the mutations also cause other defects, elsewhere in the genome. Indeed, the genome-wide genetic interactions reported here may be diagnostic of telomere-specific, or more general, chromosome stability defects caused by each mutation. We also examined interactions with a mutation affecting RPA, the central ssDNA binding protein, expected to affect general chromosome stability. The new data extend from previous analyses of telomere defective *cdc13-1* and *yku70∆* yeast strains (Addinall *et al.* 2011).

Overall, the experiments clearly show that each telomere defect shows distinct genetic interactions, with only partially conserved suppressor and enhancer interactions. This strengthens a similar conclusion drawn previously after analysis of *cdc13-1* and *yku70∆* strains (Addinall *et al.* 2011). Thus, it seems clear that there is no universal response to telomere defects, and that no single mechanism can overcome the adverse effects of telomere dysfunction. These observations in yeast are consistent with data from humans showing that mutations affecting telomere maintenance proteins cause different diseases. Individuals inheriting identical mutations can present with variable symptoms, presumably, at least in part, because other inherited mutations suppress or enhance phenotypes (Armanios *et al.* 2007; Holohan *et al.* 2014). Our work also clearly illustrates that a complex network of interactions responds to telomere defects, and that inactivation of genes that play important roles in this network (*e.g.*, *RAD9* and *EXO1*), changes the effects of other genes in the network. Consistent with this, *cdc13-1* mutants lacking *RAD9* or *EXO1* retain the ability to adapt to low-level telomere damage (Markiewicz-Potoczny and Lydall 2016).

There were many interesting patterns across the genome-wide datasets. The *exo1∆* and *nmd∆* mutations suppress most telomere defects, but are comparatively neutral, or enhance, *rfa3-313*, and are more likely to affect general DNA replication. *exo1∆* and *nmd∆* mutations reduce ssDNA levels near telomeres of *cdc13-1* strains (Holstein *et al.* 2014); this mechanism most likely explains why *exo1∆* and *nmd∆* mutations suppress the chronic and acute telomere defects examined here.

It is interesting that *rad9∆*, a checkpoint mutation affecting the yeast homolog of human 53BP1, suppresses *cdc13-1* telomere defective mutants growing with chronic telomere defects, but enhances fitness defects in nearly every other situation we tested, including *cdc13-1* strains exposed to acute telomere defects, and *stn1-13* and *rfa-313* cells growing with chronic defects. Other checkpoint mutations, most clearly *rad17∆*, *rad24∆*, and *ddc1∆*, showed different patterns. We suspect that the effects of Rad9 in the different telomere defective contexts are due to its dual roles, inhibiting ssDNA accumulation and signaling cell cycle arrest (Lazzaro *et al.* 2008).

**Figure 7:**
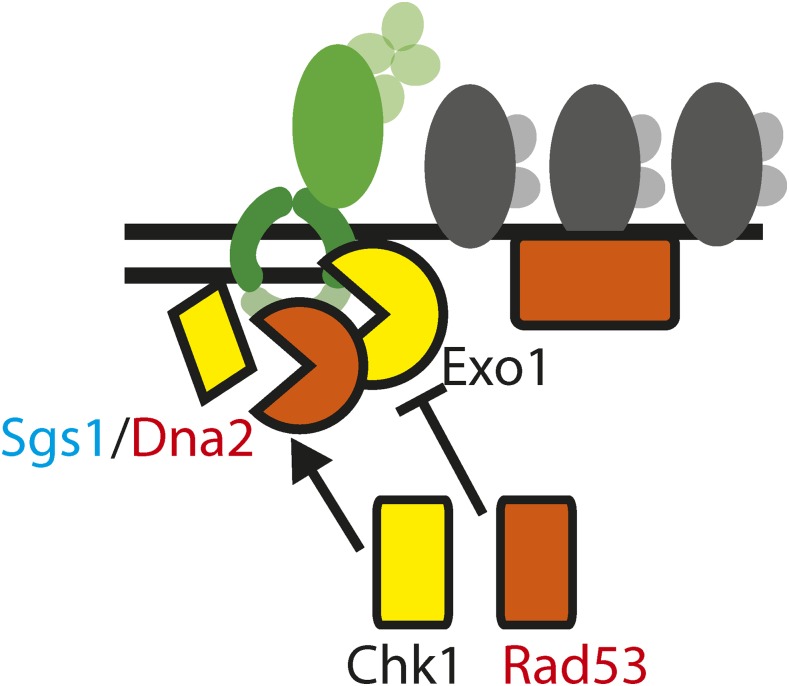
Kinase control over resection. A model of Chk1 stimulating Sgs1/Dna2 dependent resection and Rad53 inhibiting Exo1 dependent resection near uncapped telomeres of *cdc13-1* mutants.

The strong suppression of *cdc13-1* by *exo1∆ chk1∆* double mutations may be explained by the finding that Chk1 contributes to ssDNA production—a new role for Chk1 in the DNA damage response network. This effect of Chk1 is similar to that of Sgs1/Dna2, as previously reported (Ngo and Lydall 2010). We therefore propose that Chk1 stimulates Sgs1-Dna2 dependent resection (Figure 7). Consistent with this, CHK1 has been found to phosphorylate the Sgs1 homolog BLM in human cells, providing a possible mechanism for this regulation (Blasius *et al.* 2011). Additional experiments will be necessary to determine whether, in yeast, Chk1 stimulates resection by phosphorylation of Sgs1, Dna2, and/or other targets.

It is clear that *cdc13-1* and *stn1-13*, affecting two components of the CST complex, show very different genetic interactions. At face value, these differences are inconsistent with the idea that the CST complex functions as a single entity. Indeed, our favored explanation for these data are that Stn1 performs different functions to Cdc13. Along these lines, there is biochemical evidence that Stn1 can facilitate DNA replication without help from Cdc13 (Lue *et al.* 2014), and that Ten1 acts as a molecular chaperone in plants (Lee *et al.* 2016). However, the difference between *cdc13-1* and *stn1-13* could also be explained if each allele causes separations of function, and further experiments will be necessary to understand the differences.

The large volume of genetic interactions we report in this paper is potentially of value to those interested in telomere biology, DNA replication, and chromosome function. To help others explore the data in different ways, we have made them available via two complementary interactive web tools: DIXY and Profilyzer.

## Supplementary Material

Supplemental material is available online at www.g3journal.org/lookup/suppl/doi:10.1534/g3.117.042283/-/DC1.
