# Supplementary material for: Systematic Analysis of the DNA Damage Response Network in Telomere Defective Budding Yeast
Source: G3 (Bethesda). 2017 May 25;7(7):2375–89. doi: 10.1534/g3.117.042283 (PMC5499144; doi:10.1534/g3.117.042283)

# Supplementary Figure 1

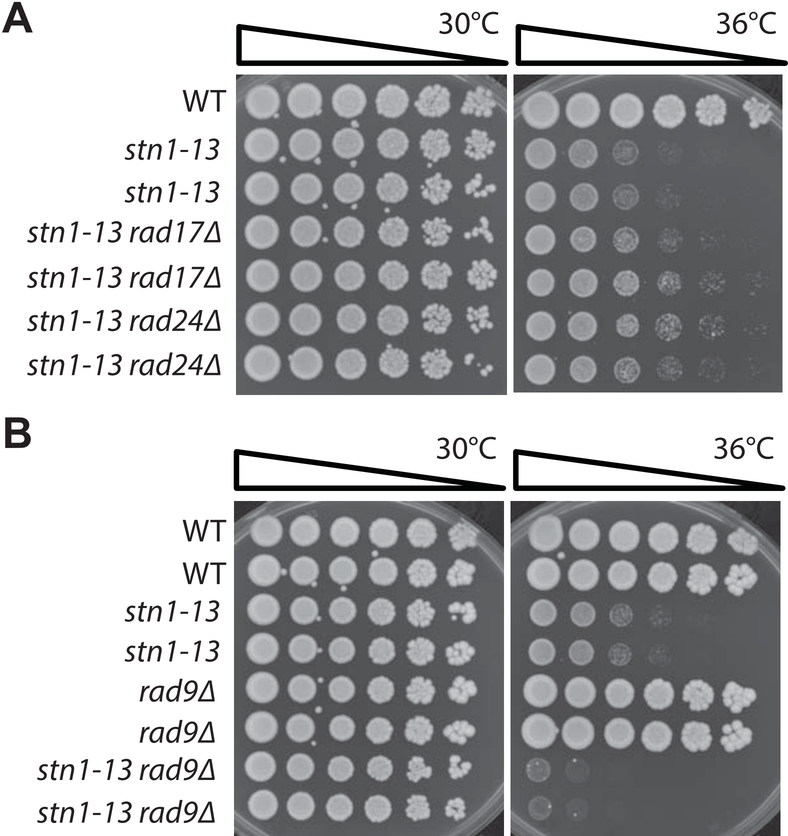

Supplementary Figure 2

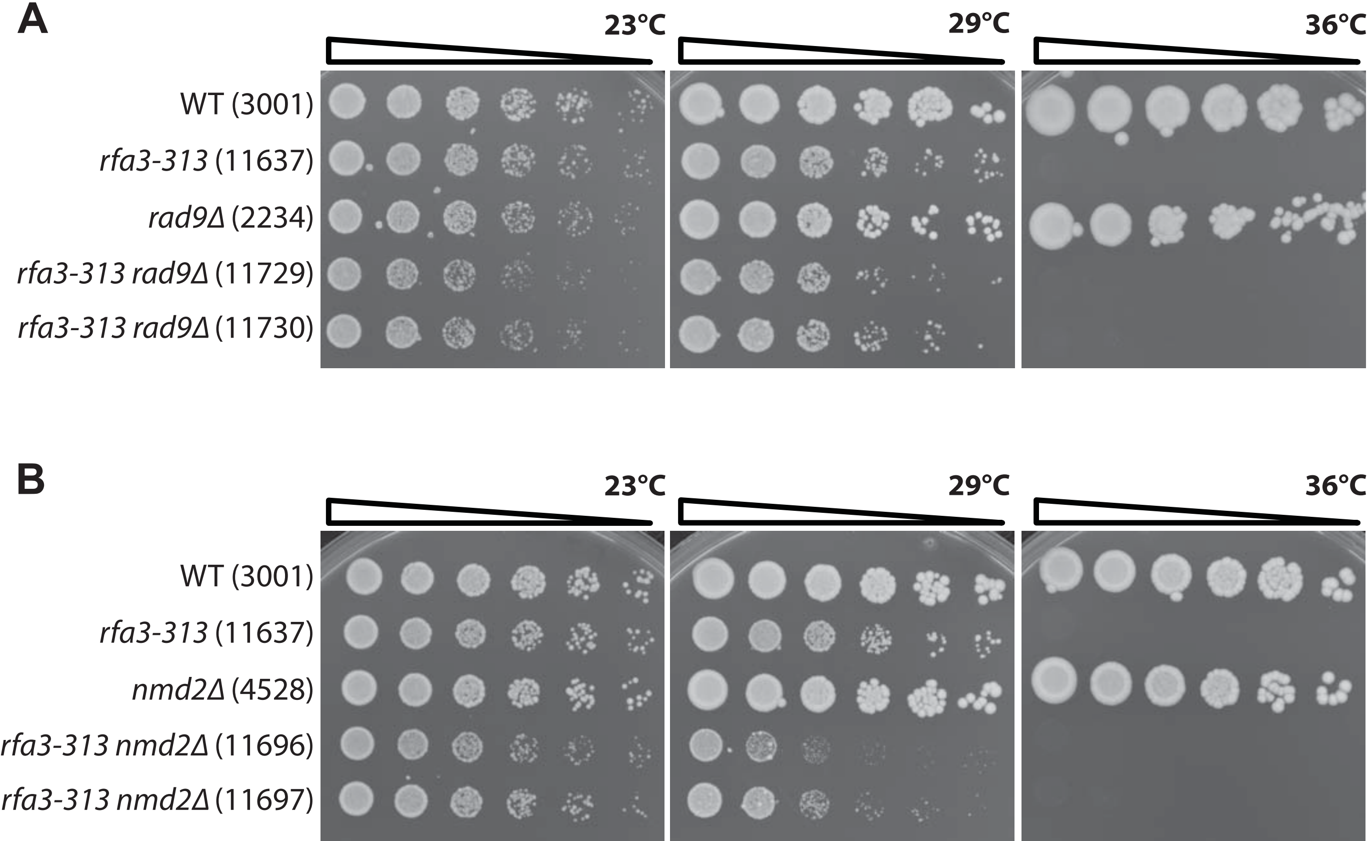

# Supplementary Figure 3

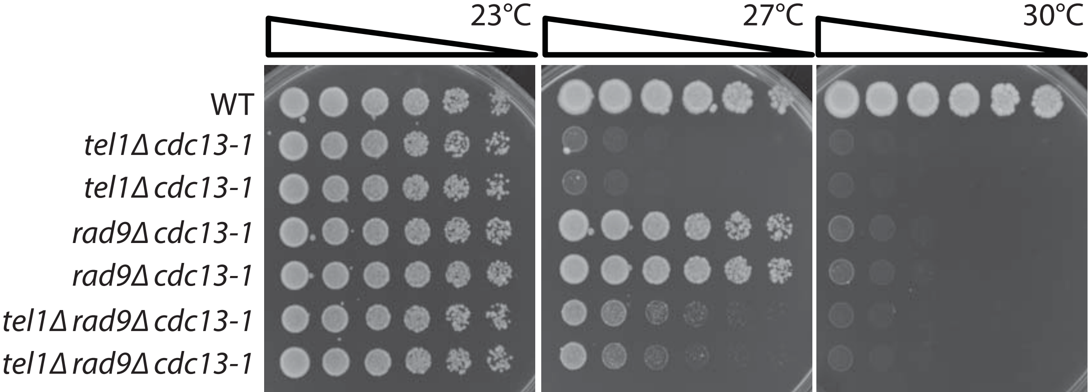

# Supplementary Figure 4

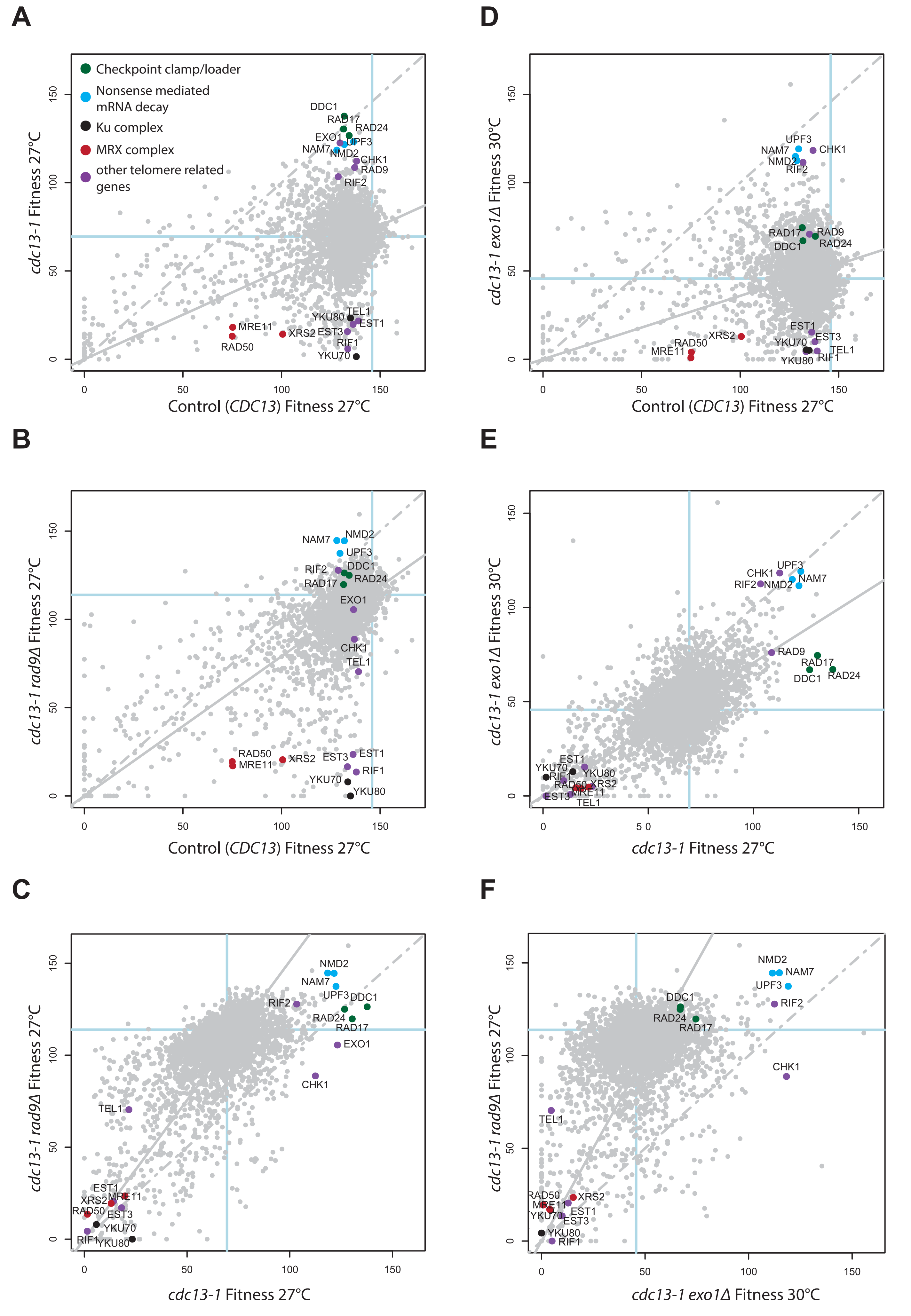

Supplement: Supplementary file 1 [file 2375FileS1.pdf]
