# Supplementary material for: Systematic Analysis of the DNA Damage Response Network in Telomere Defective Budding Yeast
Source: G3 (Bethesda). 2017 May 25;7(7):2375–89. doi: 10.1534/g3.117.042283 (PMC5499144; doi:10.1534/g3.117.042283)
Supplement: Supplementary file 2 [file 2375FileS2.pdf]

**Table 1**

| Strain | Genetic Background | Genotype | Related Figures |
|---|---|---|---|
| DLY640 | W303 | MATa | 4, S1, S3 |
| DLY1195 | W303 | MATα cdc13-1 | 4 |
| DLY1255 | W303 | MATa rad9::HIS3 cdc13-1 | S2 |
| DLY1256 | W303 | MATα rad9::HIS3 cdc13-1 | S2 |
| DLY1273 | W303 | MATa exo1::LEU2 | 5 |
| DLY1296 | W303 | MATa exo1::LEU2 cdc13-1 | 4 |
| DLY1543 | W303 | MATa tel1::TRP1 cdc13-1 | S3 |
| DLY1544 | W303 | MATα tel1::TRP1 cdc13-1 | S3 |
| DLY1585 | W303 | MATα rad9::KANMX | S1 |
| DLY2234 | W303 | MATa rad9::LEU2 | S1, S2 |
| DLY2787 | W303 | MATα yku70::LEU2 | 4 |
| DLY2988 | W303 | MATa cdc13-1 rad53::HIS3 sml1::KANMX | 5 |
| DLY3001 | W303 | MATα | S1, S2 |
| DLY4528 | W303 | MATa nmd2::HIS3 | S1 |
| DLY4557 | W303 | MATa cdc13-1 int | 5 |
| DLY4625 | W303 | MATa cdc13-1 int nmd2::HIS3 | 4 |
| DLY4647 | W303 | MATa cdc13-1 rad9::HIS3 | 5, S3 |
| DLY4921 | W303 | MATa cdc13-1 int exo1::LEU2 | 5 |
| DLY4922 | W303 | MATa cdc13-1 rad9::HIS3 exo1::LEU2 | 5 |
| DLY4931 | W303 | MATα cdc13-1 rad24::TRP1 | 5 |
| DLY5007 | W303 | MATα yku70::LEU2 nmd2::HIS3 | 4 |
| DLY5097 | W303 | MATa cdc13-1 exo1::LEU2 rad24::TRP1 | 5 |
| DLY5255 | W303 | MATα rad9::LEU2 nmd2::HIS3 cdc13-1 int | 4 |
| DLY5260 | W303 | MATa cdc13-1 cdc15-2 bar1::hisG | 6 |
| DLY5261 | W303 | MATa cdc13-1 cdc15-2 bar1::hisG | 6 |
| DLY5266 | W303 | MATa cdc13-1 cdc15-2 bar1::hisG exo1::LEU2 | 6 |
| DLY5386 | S288C | MATα LEU2::cdc13-1 int::HPHMX rad9::NATMX lyp1Δ can1::MFA1pr-HIS3 ura3 leu2 his3 LYS2+ | 2, S4 |
| DLY5688 | S288C | MATα LEU2::cdc13-1 int::HPHMX lyp1Δ can1::MFA1pr-HIS3 ura3 leu2 his3 LYS2+ | 1, 2, S4 |
| DLY6720 | S288C | MATα LEU2::cdc13-1 int::HPHMX exo1::NATMX lyp1Δ can1::STE2pr-his5 ura3 leu2 his3 LYS2+ | 2, S4 |
| DLY7106 | W303 | MATa chk1::HIS3 | 5 |
| DLY7108 | W303 | MATa exo2::LEU2 chk1::HIS3 | 5 |
| DLY7110 | W303 | MATa cdc13-1 chk1::HIS3 | 5 |
| DLY7112 | W303 | MATa cdc13-1 exo1::LEU2 chk1::HIS3 | 5 |
| DLY7143 | W303 | MATa cdc13-1 cdc15-2 bar1::hisG chk1::HIS3 | 6 |
| DLY7145 | W303 | MATa cdc13-1 cdc15-2 bar1::hisG exo1::LEU2 chk1::HIS3 | 6 |
| DLY7146 | W303 | MATa cdc13-1 cdc15-2 bar1::hisG exo1::LEU2 chk1::HIS3 | 6 |
| DLY7747 | W303 | MATa exo1::LEU2 nmd2::URA3 cdc13-1 | 4 |
| DLY8460 | W303 | MATa | 5 |
| DLY8767 | S288C | MATα LEU2::stn1-13::HPHMX lyp1::NATMX can1::STE2pr-his5 ura3 leu2 his3 met15 LYS2+ | 1 |
| DLY9181 | S288C | MATα LEU2::rfa3-313::HPHMX lyp1::NATMX can1::STE2pr-his5 ura3 leu2 his3 met15 LYS2+ | 1 |

| | | | |
|---|---|---|---|
| DLY9326 | S288C | MATα lyp1::HPHMX::LEU2::NATMX can1::STE2pr-his5 ura3 leu2 his3 met15 LYS2+ | 1 |
| DLY9866 | W303 | MATa rad9::LEU2 cdc13-1 | 4, S3 |
| DLY11098 | W303 | MATα stn1-13 | S1 |
| DLY11099 | W303 | MATα stn1-13 | S1 |
| DLY11100 | W303 | MATα stn1-13 rad9::LEU2 | S1 |
| DLY11101 | W303 | MATa stn1-13 rad9::LEU2 | S1 |
| DLY11102 | W303 | MATα stn1-13 rad24::TRP1 | S1 |
| DLY11103 | W303 | MATa stn1-13 rad24::TRP1 | S1 |
| DLY11104 | W303 | MATa stn1-13 rad17::TRP1 | S1 |
| DLY11105 | W303 | MATα stn1-13 rad17::TRP1 | S1 |
| DLY11215 | W303 | MATα nmd2::URA3 stn1-13 | 4 |
| DLY11216 | W303 | MATa stn1-13 | 4 |
| DLY11637 | W303 | MATa rfa3-313::KANMX | S2 |
| DLY11696 | W303 | MATa rfa3-313::KANMX nmd2::HIS3 | S2 |
| DLY11697 | W303 | MATα rfa3-313::KANMX nmd2::HIS3 | S2 |
| DLY11729 | W303 | MATα rfa3-313::KANMX rad9::HIS3 | S2 |
| DLY11730 | W303 | MATα rfa3-313::KANMX rad9::HIS3 | S2 |
| DLY11182 | W303 | Matα rad9::HIS3 tel1::TRP1 cdc13-1 | S3 |
| DLY11183 | W303 | Mata rad9::HIS3 tel1::TRP1 cdc13-1 | S3 |

Table 2

**Quantitative Fitness Analysis Screens**

| **Screen no.** | **Query Strain** | **Spotting** | **Media** | **Temperature** |
|---|---|---|---|---|
| QFA0141 | *ura3* | dilute | SDM_rhk_CTGN | 27°C, UD_X3 |
| QFA0132 | *lyp1* | concentrated | SDM_rhlk_CTGNH | 30°C, 33°C |
| QFA0140 | *cdc13-1* | dilute | SDM_rhlk_CTGH | 27°C, UD_X3 |
| QFA0142 | *rad9Δ cdc13-1* | dilute | SDM_rhlk_CTGNH | 27°C, UD_X1 |
| QFA0051 | *exo1Δ cdc13-1* | dilute | SDM_rhlk_CTGNH | 27°C, 30°C |
| QFA0136 | *stn1-13* | concentrated | SDM_rhlk_CTGNH | 33°C |
| QFA0131 | *rfa3-313* | concentrated | SDM_rhlk_CTGNH | 30°C |

**Table 3**

List of genes highlighted in the QFA screens

| Standard Name | Comples | Description from Saccharomyces Genome Database |
|---|---|---|
| Ddc1 | 911 sliding clamp | DNA damage checkpoint protein; part of a PCNA-like complex required for DNA damage response, required for pachytene checkpoint to inhibit cell cycle in response to unrepaired recombination intermediates; potential Cdc28p substrate; forms nuclear foci upon DNA replication stress |
| Rad17 | 911 sliding clamp | Checkpoint protein; involved in the activation of the DNA damage and meiotic pachytene checkpoints; with Mec3p and Ddc1p, forms a clamp that is loaded onto partial duplex DNA; homolog of human and S. pombe Rad1 and U. maydis Rec1 proteins |
| Rad24 | 911 sliding clamp | Checkpoint protein; involved in the activation of the DNA damage and meiotic pachytene checkpoints; subunit of a clamp loader that loads Rad17p-Mec3p-Ddc1p onto DNA; homolog of human and S. pombe Rad17 protein |
| Mre11 | MRX complex | Nuclease subunit of the MRX complex with Rad50p and Xrs2p; complex functions in repair of DNA double-strand breaks and in telomere stability; Mre11p associates with Ser/Thr-rich ORFs in premeiotic phase; nuclease activity required for MRX function; widely conserved; forms nuclear foci upon DNA replication stress |
| Rad50 | MRX complex | Subunit of MRX complex with Mre11p and Xrs2p; complex is involved in processing double-strand DNA breaks in vegetative cells, initiation of meiotic DSBs, telomere maintenance, and nonhomologous end joining; forms nuclear foci upon DNA replication stress |
| Xrs2 | MRX complex | Protein required for DNA repair; component of the Mre11 complex, which is involved in double strand breaks, meiotic recombination, telomere maintenance, and checkpoint signaling |
| Nam7 | Nonsense Mediated mRNA decay | ATP-dependent RNA helicase of the SFI superfamily; involved in nonsense mediated mRNA decay; required for efficient translation termination at nonsense codons and targeting of NMD substrates to P-bodies; binds to the small ribosomal subunit via an interaction with Rps26; forms cytoplasmic foci upon DNA replication stress |
| Nmd2 | Nonsense Mediated mRNA decay | Protein involved in the nonsense-mediated mRNA decay (NMD) pathway; interacts with Nam7p and Upf3p; involved in telomere maintenance |
| Upf3 | Nonsense Mediated mRNA decay | Component of the nonsense-mediated mRNA decay (NMD) pathway; along with Nam7p and Nmd2p; involved in decay of mRNA containing nonsense codons; involved in telomere maintenance |
| Yku70 | Ku heterodimer | Subunit of the telomeric Ku complex (Yku70p-Yku80p); involved in telomere length maintenance, structure and telomere position effect; required for localization of telomerase ribonucleoprotein to nucleus via interaction with the TLC1 guide RNA; relocates to sites of double-strand cleavage to promote nonhomologous end joining during DSB repair |

| | | |
|---|---|---|
| Yku80 | Ku heterodimer | Subunit of the telomeric Ku complex (Yku70p-Yku80p); involved in telomere length maintenance, structure and telomere position effect; required for localization of telomerase ribonucleoprotein via interaction with the TLC1 guide RNA; relocates to sites of double-strand cleavage to promote nonhomologous end joining during DSB repair |
| Est1 | Telomerase | TLC1 RNA-associated factor involved in telomere length regulation; recruitment subunit of telomerase; has G-quadruplex promoting activity required for telomere elongation; possible role in activating telomere-bound Est2p-TLC1-RNA; EST1 has a paralog, EBS1, that arose from the whole genome duplication |
| Est2 | Telomerase | Reverse transcriptase subunit of the telomerase holoenzyme; essential for telomerase core catalytic activity, involved in other aspects of telomerase assembly and function; mutations in human homolog are associated with aplastic anemia. |
| Est3 | Telomerase | Component of the telomerase holoenzyme; involved in telomere replication |
| Rif1 | Rap1 interacting factor | Protein that binds to the Rap1p C-terminus; acts synergistically with Rif2p to help control telomere length and establish telomeric silencing; involved in control of DNA replication; contributes to resection of DNA double strand breaks (DSBs); deletion results in telomere elongation |
| Rif2 | Rap1 interacting factor | Protein that binds to the Rap1p C-terminus; acts synergistically with Rif1p to help control telomere length and establish telomeric silencing; deletion results in telomere elongation; RIF2 has a paralog, ORC4, that arose from the whole genome duplication |
| Rad9 | | DNA damage-dependent checkpoint protein; required for cell-cycle arrest in G1/S, intra-S, and G2/M, plays a role in postreplication repair (PRR) pathway; transmits checkpoint signal by activating Rad53p and Chk1p; hyperphosphorylated by Mec1p and Tel1p; multiple cyclin dependent kinase consensus sites and the C-terminal BRCT domain contribute to DNA damage checkpoint activation; Rad9p Chk1 Activating Domain (CAD) is phosphorylated at multiple sites by Cdc28p/Clb2p |
| Chk1 | | Serine/threonine kinase and DNA damage checkpoint effector; mediates cell cycle arrest via phosphorylation of Pds1p; phosphorylated by checkpoint signal transducer Mec1p; homolog of S. pombe and mammalian Chk1 checkpoint kinase |
| Tel1 | | Protein kinase primarily involved in telomere length regulation; contributes to cell cycle checkpoint control in response to DNA damage; acts with Red1p and Mec1p to promote interhomolog recombination by phosphorylation of Hop1; functionally redundant with Mec1p; regulates P-body formation induced by replication stress; homolog of human ataxia-telangiectasia mutated (ATM) gene |
| Exo1 | | 5'-3' exonuclease and flap-endonuclease; involved in recombination, double-strand break repair, MMS2 error-free branch of the post replication (PRR) pathway and DNA mismatch repair; role in telomere maintenance; member of the Rad2p nuclease family, with conserved N and I nuclease domains; relative distribution to the nucleus increases upon DNA replication stress; EXO1 has a paralog, DIN7, that arose from the whole genome duplication |
